# Seasonal patterns of rhizosphere microorganisms suggest carbohydrate-degrading and nitrogen-fixing microbes contribute to the attribute of full-year shooting in woody bamboo *Cephalostachyum pingbianense*

**DOI:** 10.3389/fmicb.2022.1033293

**Published:** 2022-11-29

**Authors:** Lushuang Li, Tize Xia, Hanqi Yang

**Affiliations:** Institute of Highland Forest Science, Chinese Academy of Forestry, Kunming, Yunnan, China

**Keywords:** *Cephalostachyum* *pingbianense*, bamboo shooting, rhizosphere microbe, seasonal variation, microbial function

## Abstract

Compared with the ordinary single-season shooting among woody bamboos in Poaceae, the attribute of full-year shooting in *Cephalostachyum pingbianense* represents a unique shooting type or mechanism. Nevertheless, except for the overall physiological mechanism, the effect of ecological factors, especially soil microorganisms, on this full-year shooting characteristic remains unclear. In this study, 16S rRNA and ITS rRNA genes were sequenced using the Illumina platform. Our aims were to detect the seasonal changes in rhizospheric microbial communities of *C*. *pingbianense* and to discover the correlations of soil microbes with soil properties and bamboo shoot productivity. The results showed that seasonal change had no significant effect on bacterial alpha diversity, but significantly affected bacterial and fungal community structures as well as fungal richness. Among all soil properties examined, soil temperature, soil moisture and organic matter were the predominant factors affecting bacterial community diversity and structure. Soil temperature and soil moisture also significantly influenced fungal community structure, while available phosphorus had the greatest effect on fungal diversity. In each season, bacterial genera *Acidothermus*, *Roseiarcus*, and *Bradyrhizobium*, along with fungal genera *Saitozyma*, *Mortierella*, *Trichoderma*, etc., were dominant in bacterial and fungal communities, respectively. Bacterial community functions in four seasons were dominated by chemoheterotrophy, cellulolysis, and nitrogen fixation. Saprotrophic fungi occupied a high proportion in soil samples of all seasons. In addition, correlation analysis revealed that the bamboo shoot productivity was positively correlated with multiple microbial taxa involved in carbon and nitrogen cycles. It is proposed that highly abundant microbes involved in carbohydrate degradation and nitrogen fixation in the rhizosphere soil may contribute to the attribute of producing bamboo shoots all year round in *C*. *pingbianense*. This study is among the few cases revealing the connection between bamboo shooting characteristics and soil microorganisms, and provides new physiological and ecological insights into the forest management of woody bamboos.

## Introduction

Bamboo is an evergreen plant with the feature of rapid reproduction, fast growth, rich microbial diversity and high commercial interest (Li et al., 2020; Yuan et al., 2021; Bai et al., 2022). Under natural conditions, most bamboo species rely on vegetative reproduction, that is, the propagation of rhizome bud germination and bamboo shoot growth, due to the scarcity of flowering and seeding. Therefore, bamboo shooting is a crucial link for the individual growth and bamboo forest productivity of shoots and timber, which is the base of the bamboo industry. Almost all known woody bamboos are single-shooting, lasting 3 to 4 months. *Cephalostachyum pingbianense* (Hsueh and Y. M. Yang *ex* Yi et al.) D. Z. Li and H. Q. Yang, an endemic bamboo of southeastern Yunnan Province, China, is known as the only bamboo that produces bamboo shoots all year round in the wild and represents a unique shooting type among woody bamboos in the world (Yang et al., 2008; Zheng et al., 2018). The properties of tasty edible shoots and year-round production make *C*. *pingbianense* a rare and valuable bamboo resource. In a recent study, we explored the molecular regulatory mechanism of the full-year shooting characteristic (Li L. S. et al., 2022). It is an interesting and important topic whether this full-year shooting property is related to rhizosphere organisms, as numerous studies have demonstrated that rhizosphere conditions had a noticeable impact on plant growth and yield (e.g., Fan et al., 2021; Hakim et al., 2021; Lee et al., 2022).

The rhizosphere is described as the soil area surrounding the plant root and is affected directly by root growth activities (Hinsinger et al., 2009). This narrow region is highly complex and is considered one of the most dynamic interfaces on Earth (Bano et al., 2021). As an important component of the rhizosphere, microorganisms form the structure of rhizosphere microbial population and greatly influence the interaction between plant and soil environment (Siddharthan et al., 2022). Recent studies suggested that multiple soil ecological processes critical to plant growth were driven and maintained by microbes, and thus engineering improvement of soil microbes can largely increase plant productivity (Shi et al., 2020a; Fan et al., 2021). Generally, the primary microbial mechanisms that promote plant growth are recognized as the enhancement of nutrient absorption, hormone production, pathogen protection, and tolerance to abiotic stresses (Schlaeppi and Bulgarelli, 2015; Backer et al., 2018; Phour et al., 2020). It was reported that the bacterial genus *Flavobacterium*, *Bacillus* and *Stenotrophomonas* might affect the flowering time of Moso bamboo (*Phyllostachys edulis*) by altering the effective utilization of nitrogen (Yuan et al., 2021). Microbial inoculations, like arbuscular mycorrhizal fungi and phosphate solubilizing bacteria, could significantly raise nitrogen, phosphorus and potassium concentrations in bamboo shoot, rhizome and root, and increase the dry weight of seedlings in *Dendrocalamus strictus* (Roxb.) Nees. (Muthukumar and Udaiyan, 2006). Supplementation of nitrogen-fixing microbes and vesicular-arbuscular mycorrhizae also significantly enhanced the growth parameters, such as leave number and internodular length in seedlings of *Dendrocalamus asper* and four *Bambusa* species (Singh et al., 2020). In addition, several researches have also revealed the prospective role of soil microbes in boosting the phytoremediation of contaminated soils in bamboo forests (Zhang et al., 2020; Bian et al., 2021; Fuke et al., 2021). The above studies suggested that soil microbe was a vital factor for the promotion of nutrients, growth and health of the bamboo ecosystem.

Soil microbial community structure and diversity are key indicators reflecting the ecological characteristics of soil microorganisms, which are influenced by a variety of factors, such as vegetational types (Xia et al., 2022), climate (Lladó et al., 2017), management practices (Sun et al., 2022) and soil physicochemical properties (Lladó et al., 2018). Generally, the diversity of soil bacterial community is significantly correlated with soil pH, being lower in acidic soil and higher in neutral soil (Tecon and Or, 2017). Meanwhile, lands with high soil organic matter content possess rich microorganisms because most soil microbial communities rely on organic matter decomposition for energy and material supply (Delgado-Baquerizo et al., 2016). Also, microbial communities are dramatically influenced by seasonal changes. Seasonal cycles may affect microbial community composition by altering soil temperature and moisture or carbon and nitrogen availability for soil microorganisms (Kaiser et al., 2010; Sierra et al., 2015). Recent studies suggested that the invasion of Moso bamboo resulted in significant changes in the structure and diversity of microbial communities in native plant soils (Tian et al., 2020; Li S. Y. et al., 2022). The intercropping could markedly affect the rhizosphere soil bacterial and fungal community structures of Moso bamboo plantations (Zhang et al., 2019). However, research on seasonal changes of soil microbial communities in native bamboo forests has not been reported. On the other hand, although both soil fungal and bacterial communities are affected by environmental perturbations, they show different responses to these changes (Kaisermann et al., 2015).

The shooting of bamboo is a consequence of the germination and growth of rhizome buds, which is the basis of the bamboo food and timber industry. However, there is almost a gap in the knowledge on the relationship between bamboo shoot productivity and soil microbes. *Cephalostachyum pingbianense* has the characteristic of producing shoots in four seasons, and we speculate that this high productivity may be associated with soil microbes. In this study, we performed high-throughput sequencing of ITS rRNA and 16S rRNA genes from soil samples, determined some key soil physicochemical properties and investigated the shoot productivity of *C*. *pingbianense*. Our aims were: 1) to explore the seasonal characteristics and key impact factors on the structure and diversity of soil microbial communities in *C*. *pingbianense* rhizosphere, and 2) to assess potential correlation between bamboo shoot productivity and soil microbes. The above results will not only help us understand the response of bamboo forest soil microbes to environmental changes, deepen our knowledge of soil microbes in regulating bamboo shoot productivity, but also provide us a fresh ecological insight into the management of bamboo forest soil.

## Materials and methods

### Experimental site

The study site was situated in Dawei Mountain National Nature Reserve (DMNNR, 22°54’N, 103°42’E), Yunnan Province, China. The area is under a tropical monsoon climate, with a rainy season from May to October and a dry season from November to next April. The annual temperature is 15.2 ~ 22.9°C and the annual precipitation is 1,262 ~ 2,200 mm (Wang et al., 2018). The soil in the region is classified as yellow soil based on the Genetic Soil Classification of China. The arbor layer of *C*. *pingbianense* forest consists mainly of *Lithocarpus*. Three 30 m × 30 m sampling plots were established as replicates. Each plot contained five subplots, including one large plot of 8 m × 8 m and four small plots of 5 m × 5 m, and each subplot was separated by more than 3 m. A total of 10 clumps of vigorous bamboos were numbered and labeled in the 8 m × 8 m sample plot, and 30 bamboo clumps were marked in three replicates as sample clumps for the later investigation of emerged bamboo shoot number. These four small plots were prepared to collect soil samples in different seasons.

### Soil sample collection and bamboo shoot productivity investigation

Soil samples were collected in July 2020 (summer), October 2020 (fall), January 2021 (winter) and April 2021 (spring). Our sampling in reserve was approved by the Pingbian Sub-bureau of DMNNR. For each sampling, five bamboo clumps in a small plot were randomly selected and dug out. After shaking off the loosely bound soil, the tightly adhered soils were collected and used as rhizosphere soil. The fresh soils were mixed and sieved (mesh size 2 mm) and then divided into two parts: one part was stored at −70°C for soil microbiome analysis, and the other part was air-dried for chemical property analysis. In addition, the emerged bamboo shoots of *C*. *pingbianense* were also counted. In July and October 2020, as well as January and April 2021, the number of bamboo shoots emerged from the sample clusters was surveyed every 10 days, with the shoot tip 1 cm ~ 2 cm above the ground as the standard for shoot emergence.

### Soil physiochemical properties determination

While the soil samples were being collected, soil temperature (ST) and soil moisture (SM) in the rhizome distribution area of *C*. *pingbianense* were measured by a portable soil temperature and moisture recorder (Tr-6, SHUNKEDA, Beijing). Soil moisture herein refers to soil volumetric water content, that is, the volume fraction of water per unit of total soil volume. A portion of the collected soil samples were dried, ground and sifted to determine soil PH, organic matter, nitrogen, phosphorus and potassium levels. Soil pH was determined with a glass electrode at a 2.5:1 water: soil ratio. Soil organic matter (OM) was determined using the potassium dichromate volumetric method. Soil total nitrogen (TN) was determined using the Kjeldahl nitrogen method. Soil total potassium (TK) and soil available potassium (AK) were determined using the atomic absorption photometry method. Soil hydrolyzable nitrogen (HN) was determined using the alkaline hydrolysis diffusion method. Soil available phosphorus (AP) was determined using the hydrochloric acid-sulfuric acid extraction method (Wang et al., 2022).

### DNA extraction, PCR amplification and MiSeq sequencing

The microbial community DNA was extracted from 0.5 g of fresh soil for each sample by using the FastDNA™ SPIN Kit for Soil (MP Biomedicals, Santa Ana, CA, United States). DNA quality was examined using a NanoDrop 2000 spectrophotometer (Thermo Fisher Scientific, Wilmington, DE, United States). To identify the characteristics of soil bacterial communities, the V3-V4 variable region of the 16S rRNA gene was amplified with primers 338F (5'-ACTCCTACGGGAGGCAGCAG-3′) and 806R (5′-GGACTACHVGGGTWTCTAAT-3′) (Zeng and An, 2021). The PCR reaction system (20 μL) for bacteria included 4 μL of 5 × FastPfu buffer, 2 μL of 2.5 mM dNTPs, 0.8 μL of each primer (5 μM), 0.4 μL of *TransStart FastPfu* DNA Polymerase (TransGen, Beijing, China), 0.2 μL of BSA and 10 ng of Template DNA. Meanwhile, to identify the characteristics of fungal communities, the internal transcribed spacer 1 (ITS1) region of the ribosomal RNA gene was amplified using primers ITS1F (5′-CTTGGTCATTTAGAGGAAGTAA-3′) and ITS2R (5′-GCTGCGTTCTTCATCGATGC-3′) (Tauro et al., 2021). The PCR reaction system (20 μL) for fungi included 2 μL of 10 × buffer, 2 μL of 2.5 mM dNTPs, 0.8 μL of each primer (5 μM), 0.2 μL of *TaKaRa rTaq* DNA Polymerase (Takara, Shiga, Japan), 0.2 μL of BSA and 10 ng of Template DNA. Bacterial and fungal PCR amplifications were performed using an ABI GeneAmp® 9,700 (Applied Biosystems, Foster City, CA, United States), and the following conditions were used: 95°C for 3 min, 35 cycles at 95°C for 30 s, 55°C for 30 s, 72°C for 45 s, and a final extension at 72°C for 10 min. The PCR product qualities were detected by 2% agarose gel electrophoresis, and qualified products were used for DNA library construction using the NEXTflex® Rapid DNA-Seq Kit (Bioo Scientific, Austin, Texas, United States). Then, sequencing was performed on an Illumina MiSeqPE300 Platform (Illumina, San Diego, CA, United States) by Majorbio Bio-pharm Technology Co., Ltd. (Shanghai, China). The raw sequencing data were deposited in the NCBI Sequence Read Archive (SRA) under the BioProject number PRJNA806496.

### Data analysis

The FLASH (v1.2.11) software was used for raw reads splicing (Magoč and Salzberg, 2011) and the fastp (v0.19.6) software was used for quality control (Chen et al., 2018). Operational taxonomic units (OTUs) were classified using the Uparse (v7.0) software based on the similarity of 97% (Edgar, 2013), and the taxonomic assignment of representative OTUs was performed by the RDP classifier (Wang et al., 2007). During this process, the 16S data were aligned with the Silva database (Release138)^1^ and the ITS data were aligned with the Unite database (Release 8.0).^2^

Before calculating the diversity indices, bacterial and fungal OTU tables were rarified to the minimum reads per sample, respectively. Soil microbial community alpha diversity was determined using the Chao1 and Shannon indexes, and one-way analysis of variance (ANOVA) was used to determine whether there was a statistical difference in the diversity index among samples. Furthermore, ANOVA was also utilized to analyze the significance of soil properties as well as bamboo shoot numbers in different seasons. Based on the Bray-Curtis distance algorithm, principal co-ordinate analysis (PCoA) was used to analyze the beta diversity of microbial communities. An analysis of similarities (ANOSIM) test was used to compare within-and between-group similarity.

Venn diagrams were constructed to reveal the common or unique OTUs among samples of different seasons. According to the taxonomic information, bacterial and fungal community compositions were calculated at the phylum and genus levels. Linear discriminant analysis (LDA) effect size (LEfSe) was applied to determine the species with significant differences among samples (Segata et al., 2011), with the LDA score set to 3.5 and the taxa set from phylum to genus.

FAPROTAX was used to predict bacterial ecological functions (Louca et al., 2016) and BugBase was used to predict the bacterial phenotypes (Ward et al., 2017). The Kruskal-Wallis rank test was performed to analyze the difference in predicted results among groups. Functional profiling of soil fungal community was predicted based on FUNGuild (Nguyen et al., 2016). Redundancy analysis (RDA) was conducted to determine the relationship between microbial community structure and selected soil properties. The correlations of microbial taxa (genus level) with soil properties and bamboo shoot number were assessed by calculating Spearman’s rank correlation coefficient.

## Results

### Soil properties and emerged bamboo shoot number

**Figure 1:**
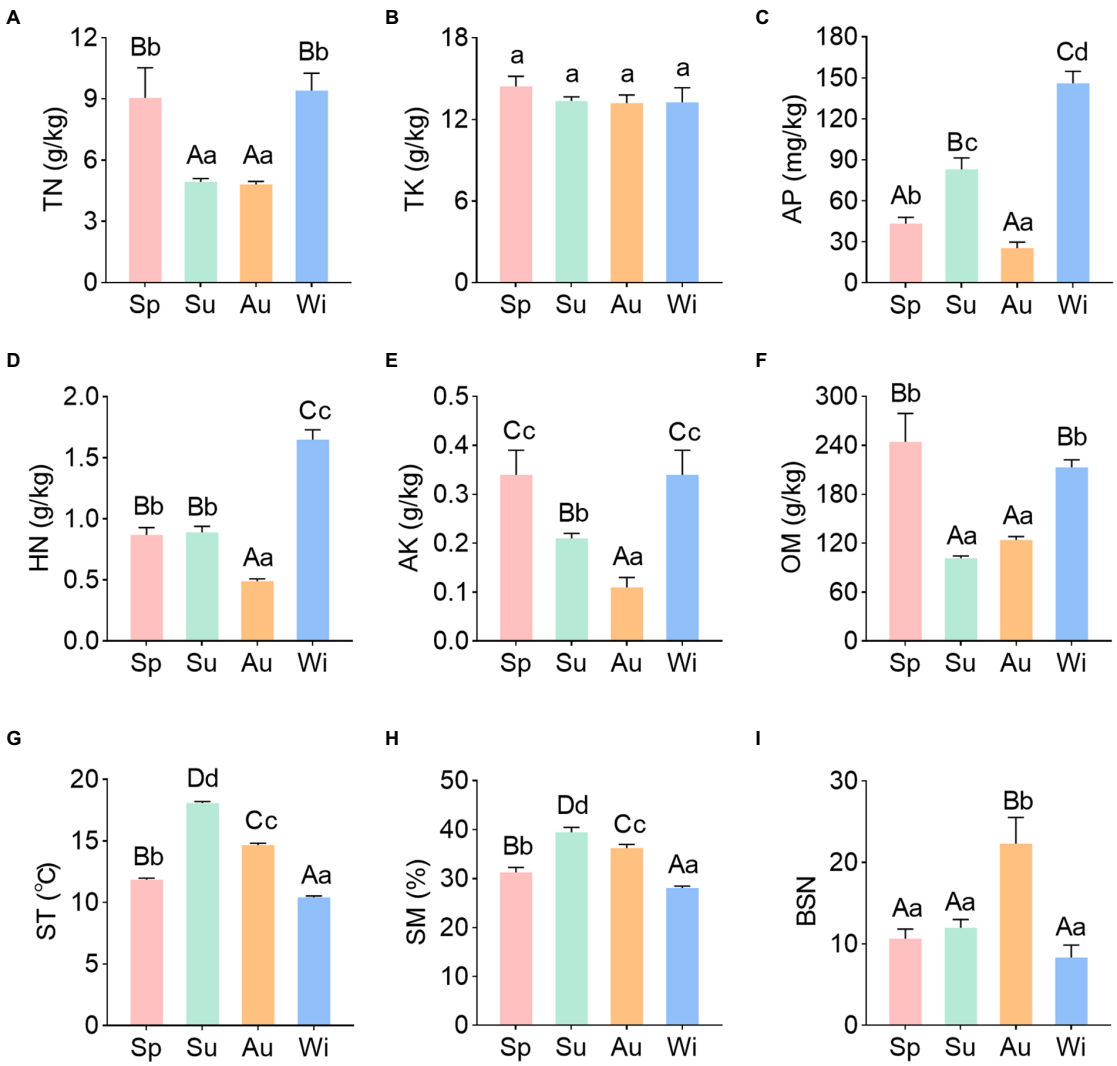
Soil physicochemical properties **(A–H)** and bamboo shoot number **(I)** of *C*. *pingbianense* in the four seasons. Different uppercase and lowercase letters in each subgraph indicated significant differences at *p* < 0.01 and *p* < 0.05 levels respectively, and error bars represented standard deviation (SD). TN, total nitrogen; TK, total potassium; AP, available phosphorus; HN, hydrolyzable nitrogen; AK, available potassium; OM, organic matter; ST, soil temperature; SM, soil moisture; BSN, bamboo shoot number; Sp, spring; Su, summer; Au, autumn; Wi, winter.

Soil physicochemical properties of *C*. *pingbianense* rhizosphere in four seasons were determined and analyzed. The rhizosphere soil was acidic and there was no significant difference in soil pH between seasons (Supplementary Figure S1). Soil TN and OM contents in summer and autumn were significantly lower than those in spring and winter (*p* < 0.01). In contrast, soil TK content did not differ significantly among samples of different seasons. Soil HN and AK contents were significantly lower in autumn than in other seasons (*p* < 0.01), and the AP content also showed the lowest value in the autumn sample. The ST and SM contents followed similar trends through the seasons, exhibiting higher levels in summer and autumn and lower levels in spring and winter (Figures 1A–H).

In addition, the numbers of emerged bamboo shoots in July, October, January and April of marked bamboo clumps were counted. Herein, these data were taken as the bamboo shooting productivity of *C*. *pingbianense* in four seasons. The result showed that there was no significant difference among the number of shoots produced in spring, summer and winter, while the shoot number in autumn was significantly higher than that in other seasons (*p* < 0.01) (Figure 1I; Supplementary Table S1).

### Soil microbial community alpha diversity and beta diversity

After quality filtering, a total of 429,954 and 791,445 effective sequences were generated from all soil samples and were classified into 3,675 bacterial OTUs and 5,119 fungal OTUs subsequently (Supplementary Table S2). Venn diagrams (Supplementary Figure S2) showed that the soil samples of four seasons had more shared bacterial OTUs than fungi, while more unique fungal OTUs were presented in the sample of each season.

**Figure 2:**
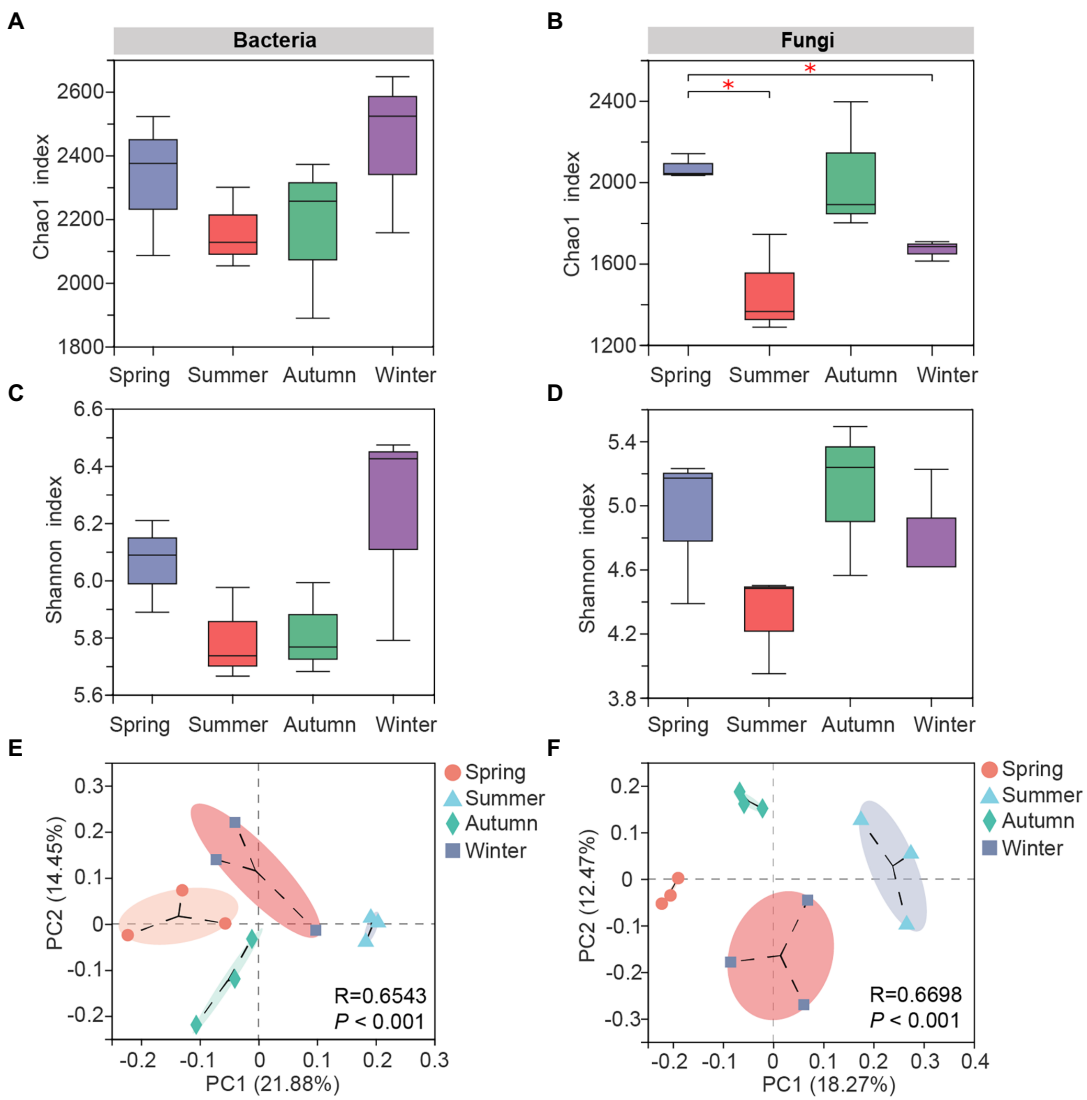
Alpha and beta diversity of rhizosphere microbes in *C*. *pingbianense* in different seasons. **(A,B)** Chao1 indexes of bacterial **(A)** and fungal **(B)** communities. **(C,D)** Shannon indexes of bacterial **(C)** and fungal **(D)** communities. **(E,F)** Principal coordinate analysis (PCoA) of bacterial **(E)** and fungal **(F)** communities based on the Bray-Curtis distance algorithm. significant differences at *p*<0.05 level

The bacterial Chao1 (Figure 2A) and Shannon indexes (Figure 2C) in soil samples of four seasons were in the descending order of winter, spring, autumn and summer, but the differences among samples were not significant, indicating the bacterial community richness and diversity in *C*. *pingbianense* rhizosphere soil did not fluctuate drastically at the seasonal scale. In contrast, the fungal Chao1 (Figure 2B) and Shannon (Figure 2D) indexes were relatively high in spring and autumn samples, and the fungal Chao1 index in spring soil showed significant differences from those in summer and winter soils (*p* < 0.05), suggesting that the fungal richness was more susceptible to seasonal changes compared to bacteria.

Results of PCoA analysis showed that soil microbial communities could be distinctly separated in different seasons, and the fungal PCoA (Figure 2F) separated samples more clearly than that of bacteria (Figure 2E). ANOSIM analysis indicated that seasonal changes significantly affected the structures of soil bacterial (*R* = 0.6543, *p* = 0.001) and fungal (*R* = 0.6698, *p* = 0.001) communities (Supplementary Figure S3). Pairwise comparisons of bacterial or fungal community among four seasons further manifested differences between diverse groups, with the R values greater than 0.25 for all sample pairs (Supplementary Table S3).

### Soil microbial community composition and function

**Figure 3:**
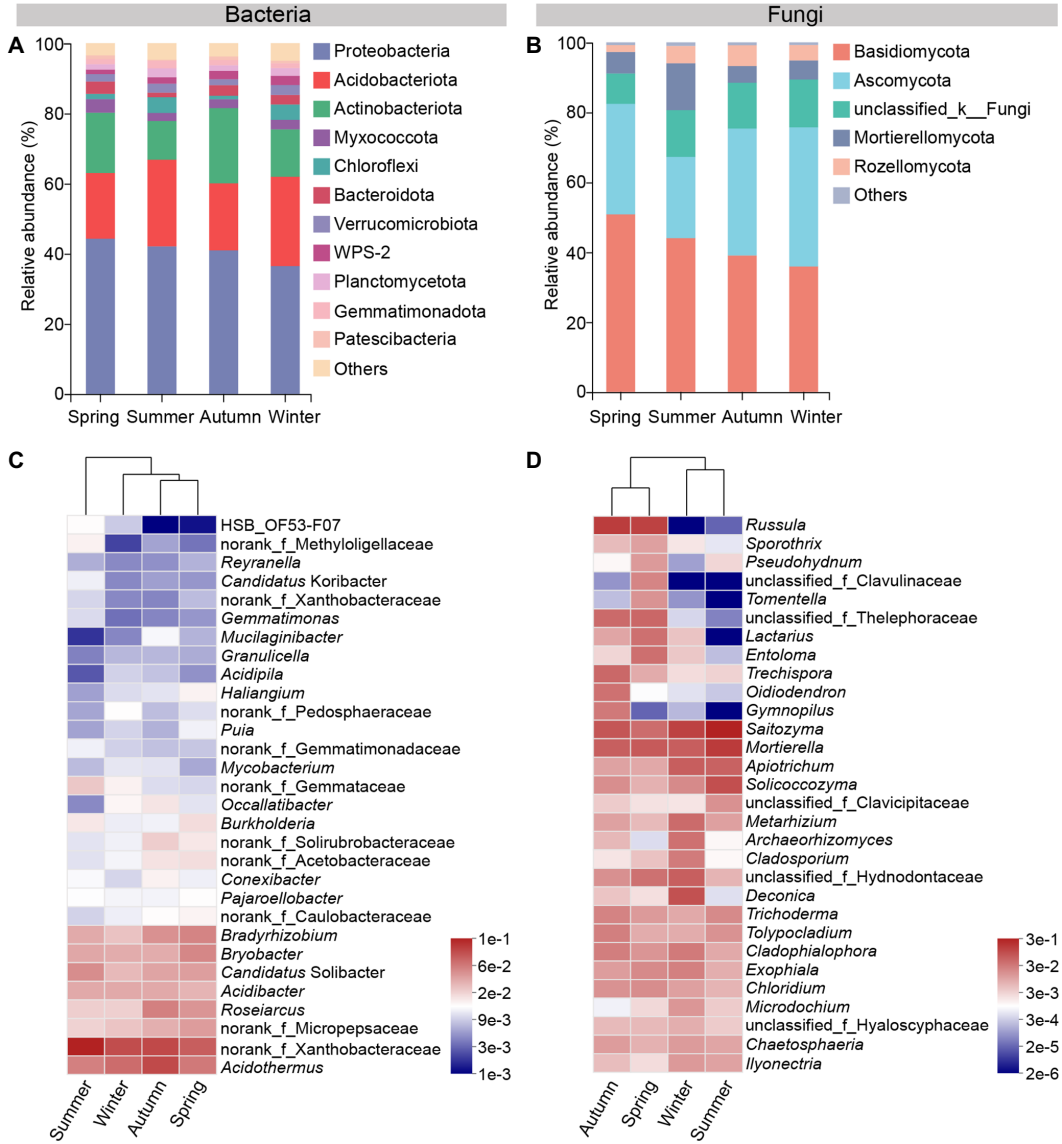
Microbial community composition in rhizosphere of *C*. *pingbianense* in four seasons. **(A,B)** Relative abundances of main bacterial **(A)** and fungal **(B)** community phyla. **(C,D)** Heatmaps showing the top 30 abundant bacterial **(C)** and fungal **(D)** genera.

Seasonal changes affect the composition of the rhizosphere microbial communities. At the phylum level, bacterial or fungal taxa with a relative abundance lower than 1% were grouped into “others.” Proteobacteria, Acidobacteriota and Actinobacteriota were the dominant soil bacterial phyla, and the three together accounted for more than 75% of bacterial communities in each season. Among them, the relative abundance of Proteobacteria decreased gradually from spring to winter. Acidobacteriota showed a higher relative abundance in summer and winter samples than that in spring and autumn, while an opposite trend was observed for Actinobacteriota (Figure 3A). At the genus level, *Acidothermus*, norank_f_Xanthobacteraceae, *Roseiarcus*, *Bradyrhizobium*, *Bryobacter* and *Candidatus* Solibacter were the dominant bacterial genera in soil samples of four seasons (Figure 3C).

As for fungi, Basidiomycota, Ascomycota, Mortierellomycota and Rozellomycota were the dominant soil fungal phyla in all seasons. Basidiomycota exhibited a downward tendency in relative abundance from spring to winter. Mortierellomycota had the highest relative abundance in the summer sample. Ascomycota and Rozellomycota showed the lowest relative abundance in the summer and spring sample, respectively (Figure 3B). At the genus level, *Saitozyma*, *Mortierella*, *Solicoccozyma*, *Trichoderma* and *Microdochium* had a high relative abundance in soil fungal communities in all seasons. What is noteworthy is that the genus *Russula* was especially enriched and dominant in spring and autumn samples (Figure 3D).

**Figure 4:**
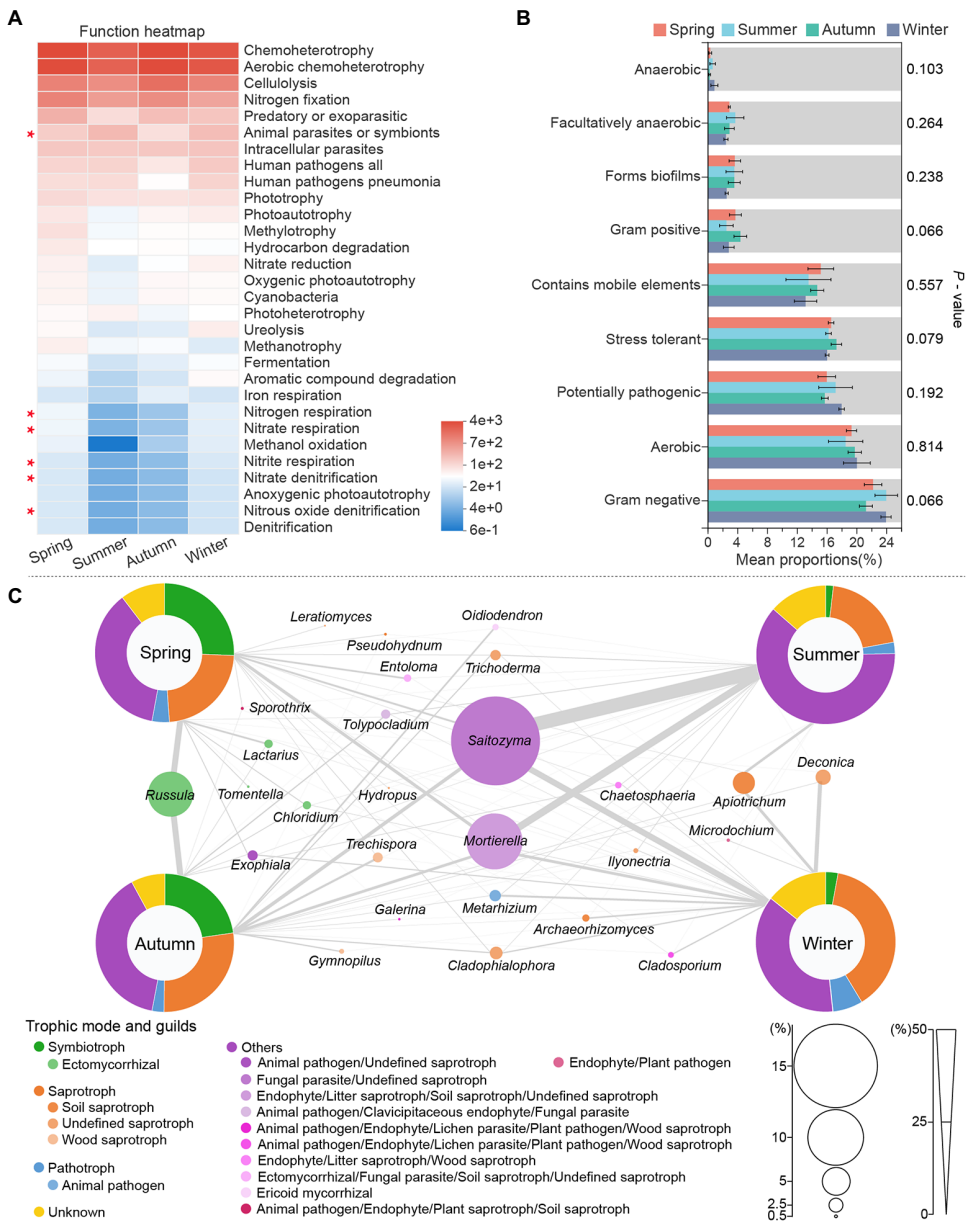
Functional prediction of rhizosphere microbes in *C*. *pingbianense* in four seasons. **(A)** Heatmap showing the top 30 bacterial ecological functions by total abundance in the samples. **(B)** Significant difference test among groups in bacterial phenotypes. **(C)** Fungal functional group composition of samples in four seasons. The size of circles in **(C)** reflected the total abundance of corresponding species in samples of all seasons, and the thickness of lines represented the abundance of that species in the sample of a certain season.

Functional predictions of the bacterial community (Figure 4A) showed that chemoheterotrophy, Aerobic chemoheterotrophy, cellulolysis and nitrogen fixation dominated the soil ecological functions in all seasons, indicating the soil bacterial community of *C*. *pingbianense* rhizosphere had a high capacity for carbohydrate conversion and biological nitrogen fixation. Furthermore, the low relative abundance of nitrate respiration, nitrite respiration and nitrous oxide denitrification suggested weak nitrification and denitrification in the bacterial community. The rhizosphere bacterial taxa were divided into nine phenotypes by the BugBase tool (Figure 4B). It was noted that the relative abundance of the same phenotypic bacteria did not differ significantly in samples of the four seasons (*p* > 0.05). In each season, however, the relative abundance varied widely across the different phenotypic bacteria. For instance, the relative abundance of Gram-negative and aerobic bacteria was much higher than that of Gram-positive and anaerobic bacteria.

Functional guild composition of the fungal community assigned by FUNGuild software was shown in Figure 4C. Saprotrophic fungi occupied a high proportion (more than 20%) in *C*. *pingbianense* rhizosphere soil of each season, including soil saprotroph (e.g., *Apiotrichum*), wood saprotroph (e.g., *Trechispora*), undefined saprotroph (e.g., *Trichoderma*) and so on. The relative abundances of symbiotrophic fungi were significantly higher in spring (25.63%) and autumn (22.70%) samples than those in summer (1.82%) and winter (2.88%), and the predominant species were ectomycorrhizal fungi, including *Russula*, *Lactarius*, *Chloridium*, etc. Pathotrophic fungi had the highest relative abundance (6.96%) in the winter sample, mainly including animal pathogens such as *Metarhizium*. Moreover, in all seasons, *Saitozyma* and *Mortierella* belonging to multiple trophic modes were dominant in soil fungal communities.

### Soil microbial taxa with statistically significant differences

**Figure 5:**
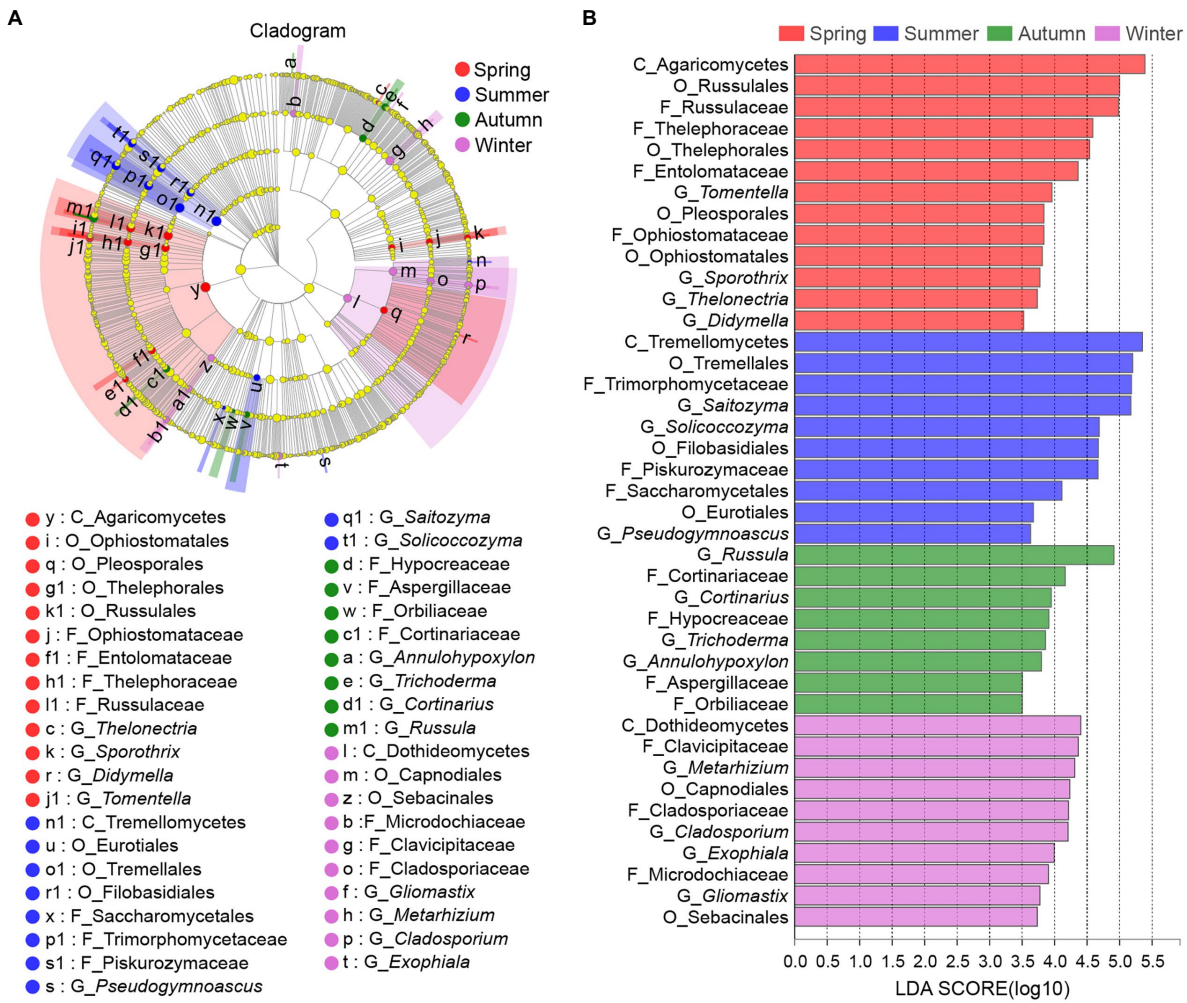
LEfSe analysis of fungal abundance in rhizosphere of *C*. *pingbianense* in four seasons. **(A)** Cladogram showing taxa with different abundance values of fungal community. **(B)** LDA bar chart of fungal community.

LEfSe was used to identify the classified microbial taxa with significant abundance differences among samples of four seasons. Here, 14 differentially abundant taxonomic clades were detected as bacterial biomarkers. The representative bacterial genera included *Pedomicrobium* (*p =* 0.044) enriched in the summer sample and *Roseiarcus* (*p =* 0.034) in the autumn sample (Supplementary Figure S4; Supplementary Table S4A). By contrast, more fungal biomarkers, namely 46 fungal clades with significant differences, were detected in this study. At the genus level, the fungal biomarkers included but were not limited to *Tomentella* (*p =* 0.015) enriched in the spring sample, *Saitozyma* (*p =* 0.033) in the summer sample, *Trichoderma* (*p =* 0.030) in the autumn sample and *Metarhizium* (*p =* 0.043) in the winter sample (Figure 5; Supplementary Table S4B).

### Correlations of soil properties with soil microbial community diversity and structure

By analyzing the correlations between selected soil properties and microbial alpha diversity indexes, we found that the bacterial Chao1 index was positively correlated with most soil properties such as OM, TN, HN, AP and AK, and negatively correlated with ST and SM. The bacterial Shannon index showed a significant positive correlation with OM and AP (*p* < 0.05), and a significant negative correlation with ST and SM (*p* < 0.05). These results suggested that OM, AP, ST and SM were important factors affecting bacterial diversity. The fungal Chao1 and Shannon indexes were positively correlated with OM and negatively correlated with AP and ST. Among examined soil properties, AP had the greatest effect on fungal diversity (Supplementary Table S5).

**Figure 6:**
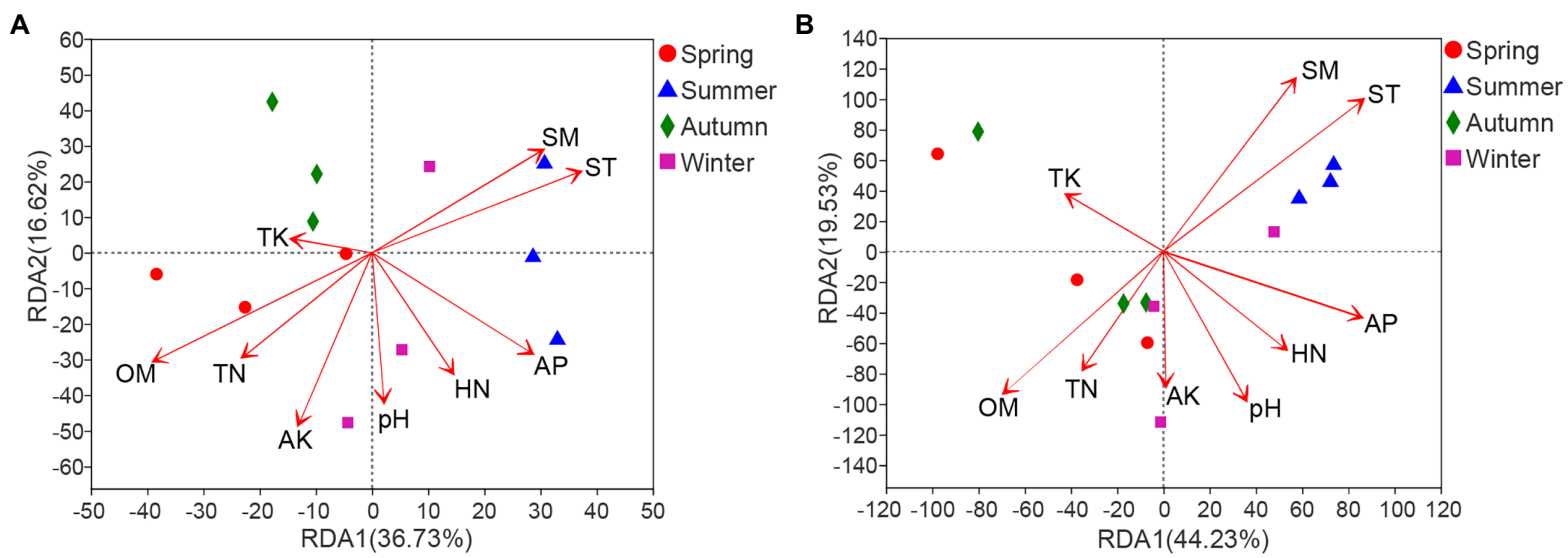
Redundancy analysis of soil properties and bacterial **(A)** or fungal **(B)** community in rhizosphere of *C*. *pingbianense*. TN, total nitrogen; TK, total potassium; HN, hydrolyzable nitrogen; AP, available phosphorus; AK, available potassium; OM, organic matter; ST, soil temperature; SM, soil moisture.

Redundancy analysis result revealed that OM had the most significant effect (*r^2^* = 0.601, *p* = 0.011) on bacterial community structure, followed by ST (*r^2^* = 0.4787, *p* = 0.043) and SM (*r^2^* = 0.4199, *p* = 0.048) (Figure 6A; Supplementary Table S6A). With regard to fungi, the community structure was also significantly influenced by ST (*r^2^* = 0.5484, *p* = 0.022) and SM (*r^2^* = 0.4972, *p* = 0.047). Although the correlation between OM and fungal community did not reach a significant level, it was the most influential soil factor on fungal community structure (*r^2^* = 0.4174, *p* = 0.071) after ST and SM (Figure 6B; Supplementary Table S6B).

### Correlations of microbial genera with soil properties and bamboo shoot number

**Figure 7:**
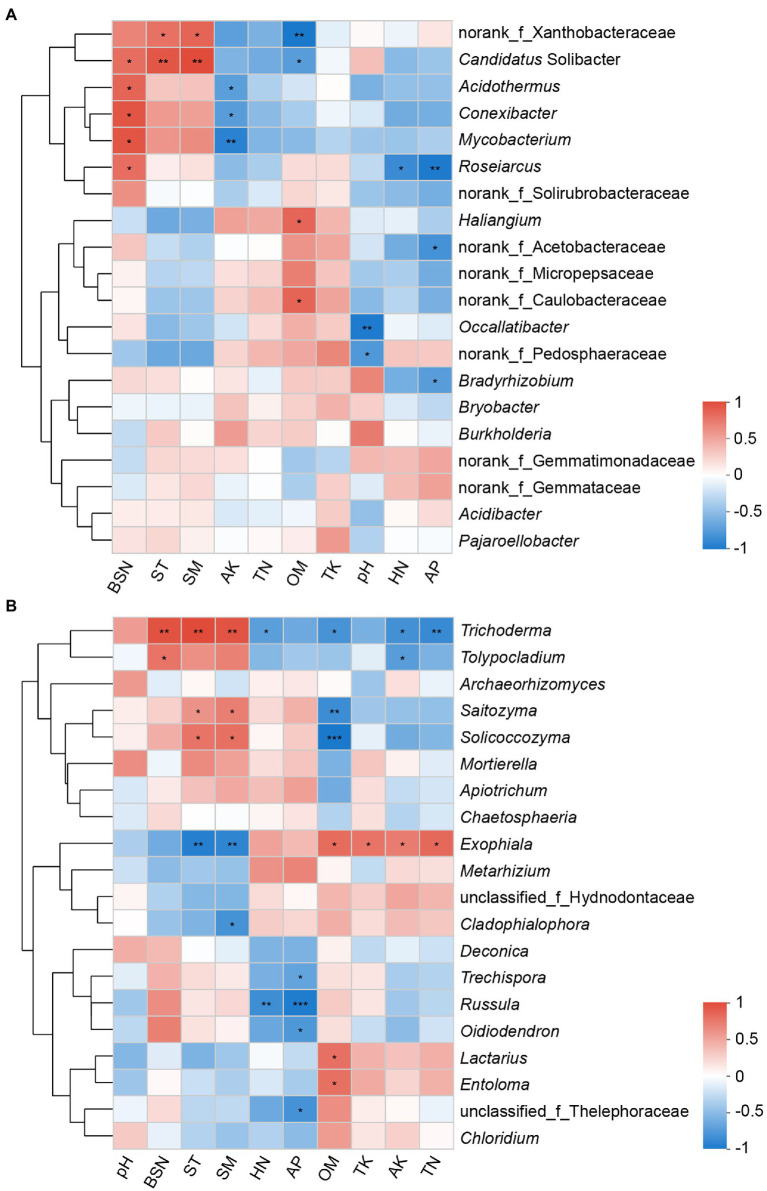
Heatmaps showing correlations between chosen factors and the first 20 genera of bacteria **(A)** and fungi **(B)**. Color depth represented the magnitude of correlation R value. *0.01 < *p* ≤ 0.05; **0.001 < *p* ≤ 0.01; ***p ≤ 0.001; BSN, bamboo shoot number; ST, soil temperature; SM, soil moisture; AK, available potassium; TN, total nitrogen; OM, organic matter; TK, total potassium; HN, hydrolyzable nitrogen; AP, available phosphorus.

Correlations of the 20 most abundant microbial genera with soil properties and bamboo shoot number were further analyzed. The abundances of bacterial genera norank_f_Xanthobacteraceae and *Candidatus*_Solibacter were found to have a significant positive correlation with ST and SM. *Candidatus*_Solibacter, *Acidothermus*, *Conexibacter*, and *Roseiarcus* were found to have a significant positive correlation with bamboo shoot number, and have a negative or significant negative correlation with OM, AP, AK, and HN (Figure 7A). With regard to the fungal community, the genera *Trichoderma* and *Tolypocladium* had a significant positive correlation with bamboo shoot number, and *Russula* had an apparent negative correlation with HN and AP. Soil temperature and moisture were positively correlated with *Trichoderma*, *Saitozyma* and *Solicoccozyma*, and negatively correlated with *Exophiala*. Additionally, the correlations between most fungal genera and OM showed an opposite pattern to those with ST and SM (Figure 7B).

## Discussion

### Seasonal variation of soil property and its influence on soil microbial communities

In this study, the SM contents of *C*. *pingbianense* rhizosphere were higher in summer and autumn than those in spring and winter, significantly affected by seasonal rainfall in the study area. The soil OM contents in summer and autumn were significantly lower than those in spring and winter. On one hand, this was likely due to the enhanced leaching of organic matter with the higher precipitation in summer and autumn (Cleveland et al., 2006; Dong et al., 2019). On the other hand, it may be because of the low ST in spring and winter resulting in weakened mineralization and relatively increased accumulation of OM (Bai et al., 2013; Brienen et al., 2015). Plants need more nutrients from the soil during their growth spurt when the soil organic carbon mineralization rate is high (Buckeridge et al., 2013). Bamboo shoots of *C*. *pingbianense* emerge in summer and peak in autumn, during which the soil OM may release large amounts of nutrients through high mineralization to meet the growing needs of bamboo shoots. The variation trend of soil TN in the four seasons was similar to that of OM. It is understandable because more than 90% of soil TN is organic nitrogen stored in the soil, and the organic nitrogen mainly concentrates in humus (Nannipieri and Eldor, 2009; Knicker, 2011). Surprisingly, in autumn, the soil HN, AP and AK contents were lower than those in other seasons. We speculated that autumn was the peak stage of bamboo shoots, and the sprouting growth consumed large amounts of nutrients in the soil. In addition, there was no significant difference in soil pH value and soil TK content among the four seasons. It was probably because these two properties were basically influenced by the soil parent material of sampling site and less affected by seasonal variations.

On the other hand, our results indicated that ST, SM, OM and AP were key factors affecting soil microbial communities of *C*. *pingbianense* rhizosphere. Soil microbial community structure can be jointly regulated by the environment and available resources (Zhao et al., 2022). The seasonal variation leads to changes in soil temperature and moisture, both of which are significant drivers of soil microbial communities (Kim et al., 2020; Shi et al., 2020b; Romero-Trigueros et al., 2021). Meanwhile, the reciprocal effect between temperature and precipitation could influence carbon cycling by regulating microbes (You et al., 2014; Taylor et al., 2017). Our results confirmed that ST, SM, and OM had the most important effects on soil microbial community structure in *C*. *pingbianense* rhizosphere. Furthermore, the soil bacterial alpha diversity was also affected by ST, SM, and OM, which was consistent with recent studies on soil microorganisms (Su et al., 2022; Zhang et al., 2022). The fungal Chao1 index was found to have a strong negative correlation with AP content. The possible explanation was that the low contents of soil-available phosphorus in spring and autumn samples may promote the symbiosis between plants and mycorrhizal fungi, thereby increasing phosphorus uptake by plants (Shi et al., 2021), while the enrichment of mycorrhizal fungi might have increased the diversity of the fungal community.

### Seasonal variation of microbial community diversity

Seasonal changes affect microbial diversity (Shen et al., 2021). In our study, the bacteria diversity in the rhizosphere soil of *C*. *pingbianense* was relatively low in summer and autumn, which was possibly associated with the high rainfall during this period. Soil moisture conditions can affect the soil pores connectivity and the adsorption capacity of soil to bacteria (Banks et al., 2003). The wetter condition is expected to expand the migration ability of dominant bacteria so that the motile species can more easily cross pore space to seek optimal conditions, thus reducing the potential of bacterial biodiversity at a macroscopic level (Wang and Or, 2010). Regarding the fungal community, the enrichment of ectomycorrhizal fungi in spring and autumn samples may probably result in the high fungal community diversity in these seasons. Although both the soil bacterial and fungal diversities were affected by seasonal changes, our results indicated that fungi were more affected. It was reported that soil bacterial and fungal communities had different adaptations to environmental perturbations, with the former being more resistant and adaptable in terms of diversity, structure and biomass (Kaisermann et al., 2015; Wang et al., 2019). Bacteria have a wider range of life than fungi and often form biofilms to resist external disturbances through extracellular polymers (Or et al., 2007), which may explain their relative stability.

### Functional profiles of the dominant species in soil microbial communities

Based on the microbial community composition analysis, three of the most dominant bacterial phyla in four seasons were Proteobacteria, Acidobacteriota, and Actinobacteriota. These bacterial phyla were also dominant taxa in other woodland soils (e.g., Cheng et al., 2021; Liang et al., 2021), indicating that these phyla had a wide ecological niche and could adapt to extensive ecological environment types. At the genus level, *Acidothermus*, norank_f_Xanthobacteraceae, *Roseiarcus*, *Candidatus* Solibacter and *Bradyrhizobium* were dominant soil bacterial genera in all seasons, most of which functioned as carbohydrate decomposers or nitrogen fixers. Among them, *Roseiarcus* and *Bradyrhizobium* belonging to Rhizobiales have the ability to nitrogen fixation (Morvan et al., 2020), while *Acidothermus* belonging to Actinobacteria has the function of degrading cellulose (Talia et al., 2012). Most members of Xanthobacteraceae are known to interact with plants and are capable of fixing nitrogen, and some species of the family can also degrade alkenes and aromatic compounds (Oren, 2014). *Candidatus* Solibacter can decompose nitrate and nitrite, and has complete enzymatic pathways (polysaccharide lyases and pectin esterases) for the degradation of cell wall polysaccharides (Ward et al., 2009; Rime et al., 2015). The functional prediction results confirmed that cellulolysis and nitrogen fixation were abundant in the bacterial community of *C*. *pingbianense* rhizosphere soil. Moreover, functions of nitrification and denitrification showed low abundances in the bacterial community. Given those two processes generally occur in neutral to slightly alkaline soil conditions (Wang et al., 2020), the acidic rhizosphere soil of *C*. *pingbianense* may inhibit both processes. Weak nitrification reduced the degree of conversion from ammonium nitrogen to nitrate nitrogen, and in contrast, the nitrogen fixation function in soil was abundant. This condition may result in high levels of ammonium nitrogen in the soil and promote the growth of bamboo shoots, as plants prefer to utilize ammonium nitrogen in the early growth stage (Li, 2008).

Similarly, in the soil fungal community of *C*. *pingbianense* rhizosphere, some fungal taxa associated with carbon metabolisms, such as Tremella yeast *Saitozyma* and *Solicoccozyma* belonging to Basidiomycota, and *Trichoderma* belonging to Ascomycota, were in high abundance in samples of various seasons. Yeast can act as a pioneer in cooperating with mold and bacteria to degrade organic matter, and promote the mineralization of soil organic carbon (Fang et al., 2021). *Saitozyma* and *Solicoccozyma* are usually isolated from habitat soil. *Saitozyma* can produce lipase (extracellular enzyme) to degrade fats, while *Solicoccozyma* has a pronounced ability to assimilate aromatic acids and low-weight aromatic compounds (Liu et al., 2015). Genus *Trichoderma* can not only secrete cellulase to degrade cellulose (Chen et al., 2021) but also participate in the dissolution of insoluble soil phosphorus, improving the ability of plants to acquire phosphorus (Bononi et al., 2020). Genus *Mortierella* and *Tolypocladium* also occupied a prominent position in the soil fungal communities. It was reported that *Mortierella* could effectively decompose organic matter and enhance the stress resistance of host plants by synthesizing arachidonic acid (Murase et al., 2012; Wani et al., 2017). Some species of *Tolypocladium* have strong antagonistic and inhibitory effects on various plant pathogenic fungi (Yu et al., 2021). The enrichment of these fungal taxa in the rhizosphere soil of *C*. *pingbianense* may promote bamboo forest growth and play a positive role in the emergence of bamboo shoots. Functional profiling of the fungal community revealed a high proportion of saprotrophic fungi in soil samples of four seasons, which may be more conducive to carbon decomposition, as carbon in rhizosphere soil is first used by saprophytic fungi (Ballhausen and de Boer, 2016). It was observed that the roots of *C*. *pingbianense* were free of mycorrhizae, and the ectomycorrhizal fungi detected probably came from the Fagaceae plants in the habitat (Toju et al., 2014). There are well-developed common mycelial networks in forest soil, which can connect host and non-host plants for carbon, nitrogen and phosphorus transfer and signal communication through shared mycelial networks (He et al., 2003; Wilson et al., 2006; Klein et al., 2016; Wang et al., 2021). As we have mentioned above, the enrichment of ectomycorrhizal fungi in spring and autumn samples may facilitate the adaptation of *C*. *pingbianense* to low phosphorus condition and enhance its access to soil phosphorus. Additionally, the fungal genus *Trichoderma* and bacterial genus *Roseiarcus* were identified as biomarkers in the autumn sample through LEfSe analysis. Although these two genera were dominant in soils of all seasons, they possessed higher abundances in the autumn sample, which may be one reason for the higher amounts of bamboo shoots in autumn compared to other seasons.

### Potential key microbes associated with the full-year shooting attribute of *Cephalostachyum pingbianense*

Soil microbes reflect soil type, environmental conditions and woodland management to some extent, and play a crucial role in soil ecosystems, especially in the regulation of carbon and nutrient cycles (Coskun et al., 2017; Prommer et al., 2020). In this research, bacterial genera *Candidatus*_Solibacter, *Acidothermus* and *Roseiarcus*, as well as fungal genera *Trichoderma* and *Tolypocladium*, were found to have a significant positive correlation with bamboo shoot productivity. At the same time, these taxa were also dominant microbes in the rhizosphere soil of *C*. *pingbianense*, and their significant roles in soil carbon and nitrogen cycles have been described in detail in the above discussion. Woody bamboos are considered the representative plant with the fastest growth speed in the world. The growth and development of shoots and young culms depend on the attached mature bamboo culms and the soil to provide adequate nutrients (Sun et al., 2019), and several studies have investigated that the stress of nutrient conditions in bamboo forests can lead to the reduction and degradation of bamboo shoots (Zhong et al., 2014; Chen et al., 2019). As *C*. *pingbianense* can produce bamboo shoots throughout the year, the sprouting of bamboo shoots and growth of young culms theoretically require a large supply of nutrients. The possible mechanism is that the humid evergreen broad-leaved forest in the upper layer provides rich litters, and the enrichments of microbes associated with carbohydrate degradation and nitrogen fixation in *C*. *pingbianense* rhizosphere accelerate the mineralization of organic matter and the fixation of available nitrogen, providing continuous and sufficient nutrients for the germination of rhizome buds and the growth of bamboo shoots. To sum up, these specific microbes may be one important ecological factor for the full-year shooting characteristic of *C*. *pingbianense*.

## Conclusion

This study focused on the seasonal variation of soil microbial communities in *C*. *pingbianense* rhizosphere and the correlations of soil microbes with soil properties and bamboo shoot productivity. Compared to the bacterial community, the diversity and structure of the fungal community were more sensitive to seasonality. The soil properties of ST, SM and OM were the predominant factors affecting the diversity and structure of the bacterial community. The fungal community structure was also markedly affected by ST and SM, while the diversity was most influenced by AP. In addition, various microbes involved in carbon and nitrogen cycles were positively correlated with bamboo shoot number and dominated in soil microbial communities in all seasons. Significant enrichment of these microbes may contribute to the attribute of full-year shooting in *C*. *pingbianense*. This paper constitutes one of the initial researches exploring the seasonal variations in the structure, diversity and function of bamboo rhizosphere microbial communities. To improve bamboo shoot and timber productivity, we believe that besides the biological characteristics of bamboo species, more attention should be paid to the effects of soil microbes in bamboo forest management.

## Data availability statement

The datasets presented in this study can be found in online repositories. The names of the repository/repositories and accession number(s) can be found in the article/Supplementary material.

## Author contributions

HY: conceptualization, writing–review and editing, and funding acquisition. LL and TX: methodology, formal analysis, and resources. LL: data curation, writing–original draft preparation, and visualization. All authors read and approved the final manuscript.

## Funding

This work was supported by the Fundamental Research Funds of the Chinese Academy of Forestry (No. CAFYBB2021SZ001) and the National Natural Science Foundation of China (No. 31870574).

## Acknowledgments

We would like to thank the editor and two reviewers for their constructive comments on this manuscript, Wei Mao (Southwest Forestry University) for improving this paper, and Linna Chen, Bin Li, and Peitong Dou for their help in laboratory work. We are also grateful to the Pingbian Sub-bureau of Dawei Mountain National Nature Reserve for the assistance in experimental sampling.

## Conflict of interest

The authors declare that the research was conducted in the absence of any commercial or financial relationships that could be construed as a potential conflict of interest.

## Publisher’s note

All claims expressed in this article are solely those of the authors and do not necessarily represent those of their affiliated organizations, or those of the publisher, the editors and the reviewers. Any product that may be evaluated in this article, or claim that may be made by its manufacturer, is not guaranteed or endorsed by the publisher.

## Footnotes

^1^ http://www.arb-silva.de

^2^ http://unite.ut.ee/index.php
