# Supplementary material for: Seasonal patterns of rhizosphere microorganisms suggest carbohydrate-degrading and nitrogen-fixing microbes contribute to the attribute of full-year shooting in woody bamboo Cephalostachyum pingbianense
Source: Front Microbiol. 2022 Nov 29;13:1033293. doi: 10.3389/fmicb.2022.1033293 (PMC9745117; doi:10.3389/fmicb.2022.1033293)
Supplement: Supplementary file 1 [file Data_Sheet_1.PDF]

Supplementary Material

1.1 Supplementary Figures

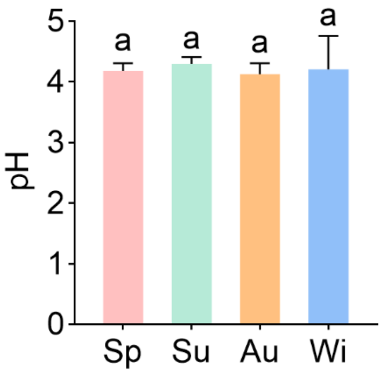

Supplementary Figure 1. Significant difference analysis of soil pH values in four seasons. Different lowercase letters indicated significant differences at $P < 0.05$ levels, and error bars represented standard deviation (SD). Sp, spring; Su, summer; Au, autumn; Wi, winter.

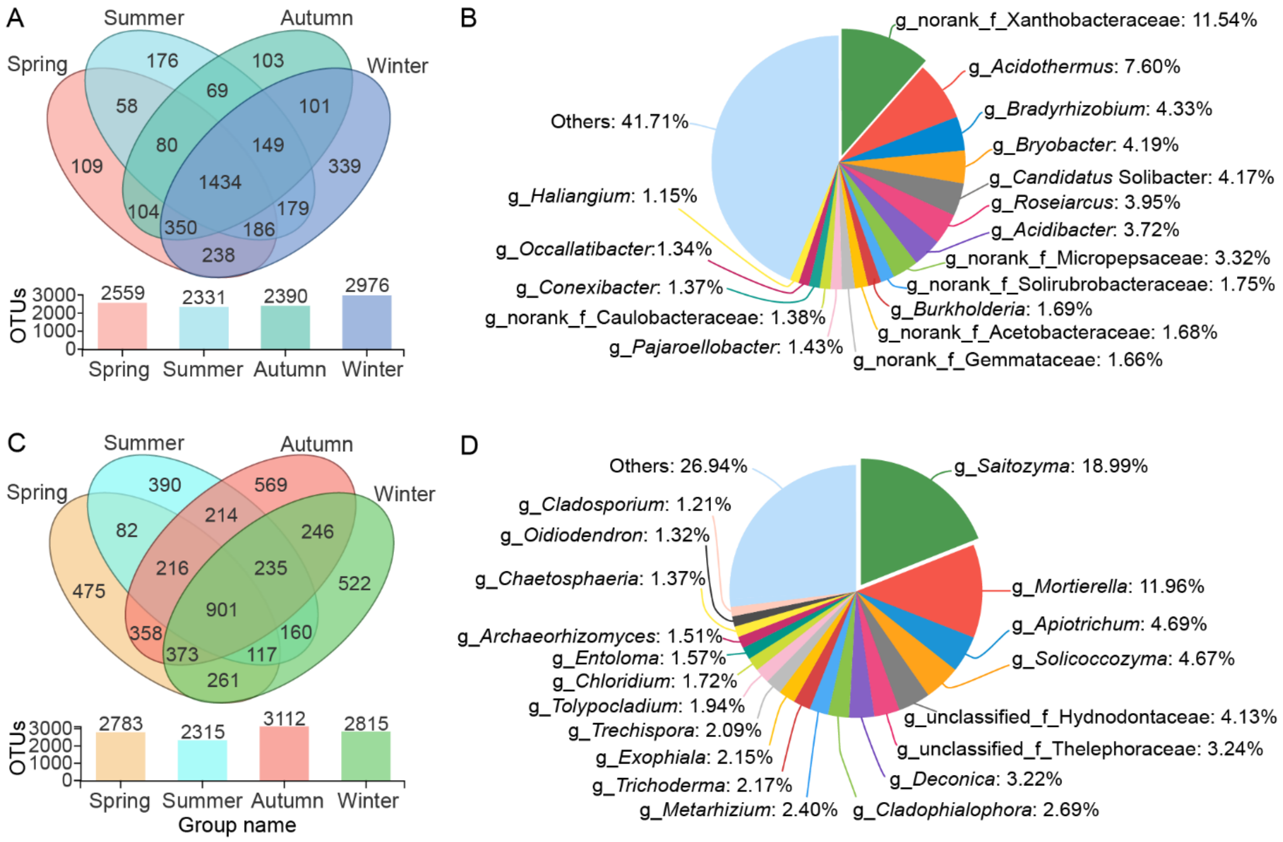

Supplementary Figure 2. Shared microbial groups in rhizosphere of *C. pingbianense* in four seasons. (A, C) Venn diagrams showing the number of shared and unique bacterial (A) and fungal (C) OTUs among samples. (B, D) Shared bacterial (B) and fungal (D) genera among samples.

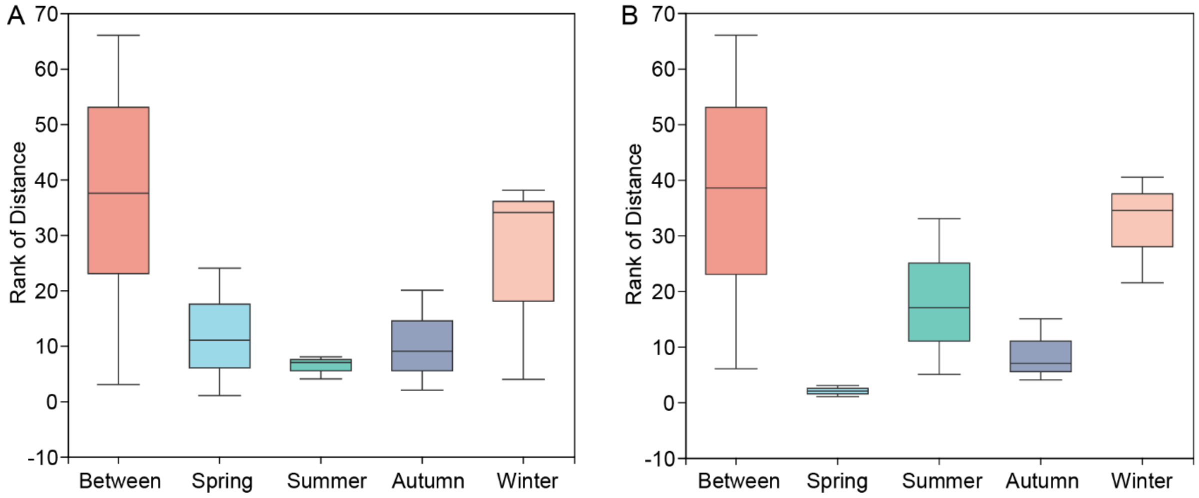

Supplementary Figure 3. Results of analysis of similarities (ANOSIM) of the soil bacterial (A) and fungal (B) communities based on the Bray-Curtis distance algorithm.

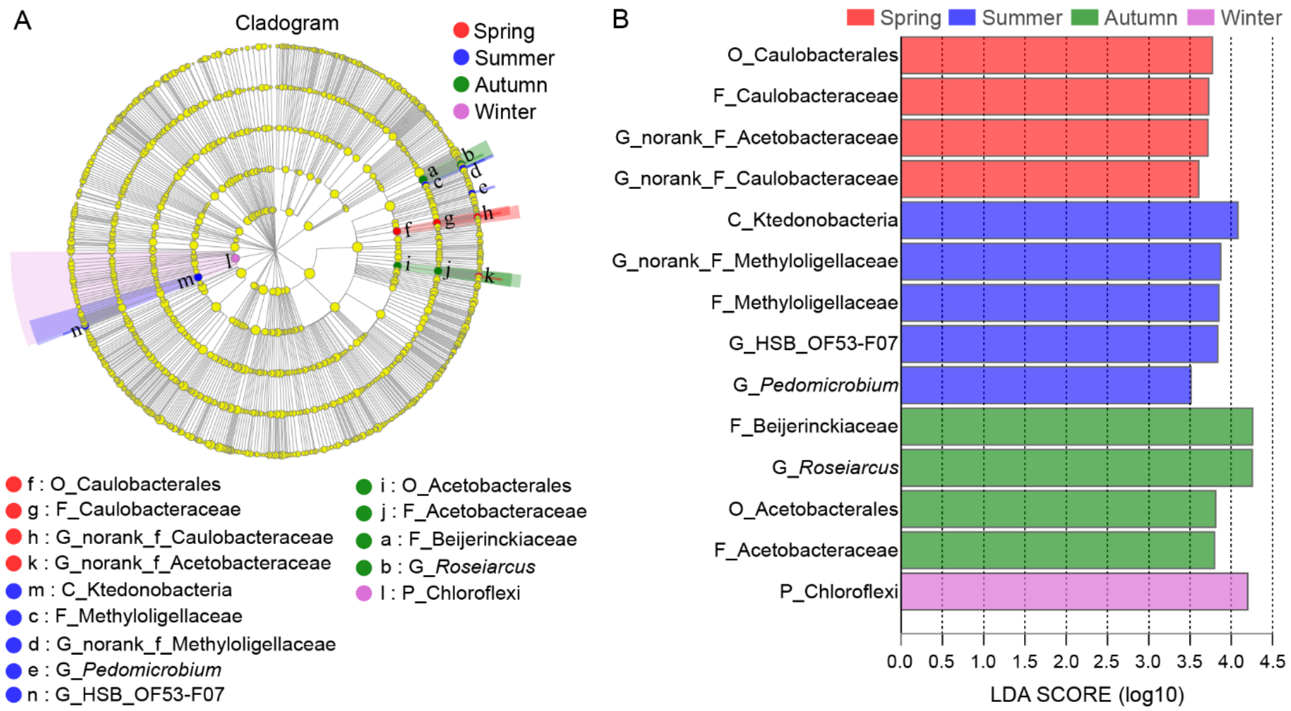

Supplementary Figure 4. LEfSe analysis of bacterial abundance in rhizosphere of *C. pingbianense* in four seasons. (A) Cladogram showing taxa with different abundance values of bacterial community. (B) Linear discriminant analysis (LDA) bar chart of bacterial community.

1.2 Supplementary Table Legends

Supplemental Table 1. Bamboo shoot number of *C. pingbianense* in different seasons.

Supplemental Table 2. The statistics of sample sequencing data.

Supplemental Table 3. ANOSIM analysis between sample pairs of soil microbial communities.

Supplemental Table 4. Classified microbial taxa with significant abundance differences among samples of four seasons.

Supplemental Table 5. Correlation between rhizosphere microbial alpha diversity and soil properties in *C. pingbianense*.

Supplemental Table 6. RDA analysis related data.
